# Pooled RT-qPCR testing for SARS-CoV-2 surveillance in schools - a cluster randomised trial

**DOI:** 10.1016/j.eclinm.2021.101082

**Published:** 2021-08-25

**Authors:** Alexander Joachim, Felix Dewald, Isabelle Suárez, Michael Zemlin, Isabelle Lang, Regine Stutz, Anna Marthaler, Hans Martin Bosse, Nadine Lübke, Juliane Münch, Marie-Annett Bernard, Kathrin Jeltsch, Burkhard Tönshoff, Niklas Weidner, Hans-Georg Kräusslich, Lena Birzele, Johannes Hübner, Patricia Schmied, Melanie Meyer-Bühn, Gibran Horemheb-Rubio, Oliver A. Cornely, Heinz Haverkamp, Gerhard Wiesmüller, Gerd Fätkenheuer, Barbara Hero, Rolf Kaiser, Jörg Dötsch, Jan Rybniker, for the B-FAST study group, Zülfü C. Cosgun, Christoph Hünseler, Jana Schönenkorb, Juliane Wurm, Florian Klein, Eva Heger, Elena Knops, Saleta Sierra-Aragón, Alina Chloé Kretschmer, Rosanne Sprute, Annelene Kossow, Martin Hellmich, Kija Shah-Hosseini, Michael Weiss, Sybelle Goedicke-Fritz, Elisabeth Kaiser, Sascha Meyer, Nastasja Seiwert, Sigrun Smola, Thorsten Pfuhl, Stefan Lohse, Anna-Kathrin Schupp, Jörg Timm, Nehle Gröne, Hellen Lesmann, Renate Bredahl, Lukas Schneble, Martin Turinsky, Christian Patry, Georg F Hoffmann, Barbara Müller, Kathleen Börner, Paul Schnitzler, Anke-Mareil Heuser, Andreas Welker, Ulrich von Both, Anna Kern

**Affiliations:** a Department of Pediatrics, University Hospital Cologne, University of Cologne, Cologne, Germany; b Institute of Virology, University Hospital Cologne, University of Cologne, Cologne, Germany; c Department I of Internal Medicine, Division of Infectious Diseases, University Hospital Cologne, University of Cologne, Cologne, Germany; d Department of General Pediatrics and Neonatology, Saarland University Homburg, Homburg, Germany; e Institute of Virology, Saarland University Homburg, Homburg, Germany; f Department of General Pediatrics, Neonatology and Pediatric Cardiology, University Children´s Hospital, University Hospital Düsseldorf, Heinrich-Heine-University Düsseldorf, Germany; g Institute of Virology, University Hospital Düsseldorf, Heinrich-Heine-University Düsseldorf, Germany; h Department of Pediatrics I, University Children's Hospital Heidelberg, Heidelberg University, Heidelberg, Germany; i Department of Infectious Diseases, Virology, Heidelberg University, Heidelberg, Germany; j Division of Pediatric Infectious Disease, Dr. v. Hauner Children's Hospital, University of Munich (LMU), Munich, Germany; k Center for Molecular Medicine Cologne, University of Cologne, Cologne, Germany; l Cologne Excellence Cluster on Cellular Stress Responses in Aging-Associated Diseases (CECAD), University of Cologne, Cologne, Germany; m German Center for Infection Research (DZIF), Partner Site Bonn-Cologne, Cologne, Germany; n Institute of Medical Statistics and Computational Biology, Faculty of Medicine and University Hospital Cologne, University of Cologne, Cologne, Germany; o Public Health Department Cologne, Cologne, Germany; p Institute for Occupational, Social and Environmental Medicine, Uniclinic RWTH Aachen University, Aachen, Germany

**Keywords:** Covid-19, SARS-CoV-2, School, Pooled testing, Surveillance, RT-qPCR

## Abstract

**Background:**

The extent to which children and adolescents contribute to SARS-CoV-2 transmission remains not fully understood. Novel high-capacity testing methods may provide real-time epidemiological data in educational settings helping to establish a rational approach to prevent and minimize SARS-CoV-2 transmission. We investigated whether pooling of samples for SARS-CoV-2 detection by RT-qPCR is a sensitive and feasible high-capacity diagnostic strategy for surveillance of SARS-CoV-2 infections in schools.

**Methods:**

In this study, students and school staff of 14 educational facilities in Germany were tested sequentially between November 9 and December 23, 2020, two or three times per week for at least three consecutive weeks. Participants were randomized for evaluation of two different age adjusted swab sampling methods (oropharyngeal swabs or buccal swabs compared to saliva swabs using a ‘lolli method’). Swabs were collected and pooled for SARS-CoV-2 RT-qPCR. Individuals of positive pooled tests were retested by RT-qPCR the same or the following day. Positive individuals were quarantined while the SARS-CoV-2 negative individuals remained in class with continued pooled RT-qPCR surveillance. The study is registered with the German Clinical Trials register (registration number: DRKS00023911).

**Findings:**

5,537 individuals were eligible and 3970 participants were enroled and included in the analysis. In students, a total of 21,978 swabs were taken and combined in 2218 pooled RT-qPCR tests. We detected 41 positive pooled tests (1·8%) leading to 36 SARS-CoV-2 cases among students which could be identified by individual re-testing. The cumulative 3-week incidence for primary schools was 564/100,000 (6/1064, additionally 1 infection detected in week 4) and 1249/100,000 (29/2322) for secondary schools. In secondary schools, there was no difference in the number of SARS-CoV-2 positive students identified from pooled oropharyngeal swabs compared to those identified from pooled saliva samples (lolli method) (14 vs. 15 cases; 1·3% vs. 1·3%; OR 1.1; 95%-CI 0·5–2·5). A single secondary school accounted for 17 of 36 cases (47%) indicating a high burden of asymptomatic prevalent SARS-CoV-2 cases in the respective school and community.

**Interpretation:**

In educational settings, SARS-CoV-2 screening by RT-qPCR-based pooled testing with easily obtainable saliva samples is a feasible method to detect incident cases and observe transmission dynamics.

**Funding:**

Federal Ministry of education and research (BMBF; Project B-FAST in “NaFoUniMedCovid19”; registration number: 01KX2021).

**Research in context**

**Evidence before this study**

We searched PubMed for articles published between Jan 1 2020 and May 1 2021, using the terms “COVID-19″ or “SARS-CoV-2″ or “SARS-CoV-2 and surveillance” and “school” or “children” or “education” or “pooled testing” to identify publications relating to surveillance strategies and transmission dynamics in educational settings. There are several screening studies that are ongoing in parallel to our study, e.g. studies using a gargling test for sampling and RT-qPCR. Several studies show a good correlation of test results based on pooled RT-qPCR compared to individual RT-qPCR testing in adults and non-school settings. There are no randomized trials investigating testing strategies in schools.

**Added value of this study**

We exploited pooled RT-qPCR testing for detection of SARS-CoV-2 in 3386 children and adolescents and 584 staff members in 14 schools. Additionally, we compared efficacy of age adjusted swab sampling methods in a randomized fashion. All classes and groups were tested two or three times per week for three consecutive weeks. Saliva samples were collected using the ‘lolli method’ in which students suck on the swab for 15 s. Collection of 21,978 swabs in children and adolescents and combining these in 2218 pooled RT-qPCRs led to identification of 36 SARS-CoV-2 positive cases.

**Implication of all the available evidence**

The data confirms the presence of a substantial number of asymptomatic prevalent SARS-CoV-2 cases in children and adolescents. This should trigger more routine screening in educational facilities especially when SARS-CoV-2 incidence rates are high in the general population. Pooled RT-qPCR testing of saliva samples appears to be a feasible approach for such settings.

Alt-text: Unlabelled box

## 1 Introduction

In response to the COVID-19 pandemic, many countries are implementing various non-pharmaceutical interventions (NPI) to contain the spread of SARS-CoV-2. Whether closing of educational institutions is an effective NPI to fight COVID-19 is debated [1,2]. School closures have major social and health side effects as well as negative consequences for the educational needs of children [3,4]. Continuous closing of schools is widening the achievement gap, primarily in children from disadvantaged backgrounds [5]. Thus, school closures should be implemented only as a very last resort.

In addition, the role of children in the transmission of SARS-CoV-2 is not fully understood [6]. Young children mainly develop mild or no symptoms. Asymptomatic infections interfere with the prediction of incidence rates in young individuals [7,8]. Adolescents aged ≥ 12 years have a higher prevalence of SARS-CoV-2 infections than younger children [9–11]. The situation is complicated by the emergence of new SARS-CoV-2 variants with altered transmission dynamics in children and adolescents [12]. Thus, better surveillance and testing strategies in educational settings are urgently needed to support decisions about school closures and re-opening strategies [13]. However, apart from screening for COVID-19 symptoms in children and adolescents, there have been little coordinated efforts to implement feasible and scalable surveillance tools in this important sector of our societies [14]. Efficacy of applied testing strategies has rarely been determined in randomised trials [13,15]. In terms of preventing SARS-CoV-2 transmission in schools, it is striking that routine SARS-CoV-2 RT-qPCR testing has received little attention so far [5]. In other settings, pooling of samples for subsequent SARS-CoV-2 detection by RT-qPCR has been demonstrated as a sensitive high-capacity diagnostic method [16,17].

Application of mass testing regimes with point of care lateral flow devices (LFD) or rapid antigen tests are increasingly adopted in schools. However, the relatively complex LFD testing process may require presence of experienced medical staff especially when applied in younger children. In addition, there are concerns regarding sensitivity and specificity of LFD systems in asymptomatic individuals [18,19].

Nasopharyngeal testing is the current standard for SARS-CoV-2 detection. However, repetitive nasopharyngeal mass-testing in children and adolescents may result in low acceptance rates and non-reliable test results when performed by parents, teachers or as a self-testing procedure. Saliva based RT-qPCR testing is an alternative approach with acceptable sensitivity and specificity [20]. Performance of these novel sampling methods in educational settings should be evaluated in prospective studies.

As part of the first national lockdown, schools in Germany closed on March 17, 2020. The gradual easing of the lockdown also included the reopening of primary and secondary schools starting in May 2020. Schools continued to operate with applied safety and hygiene-related measures until a significant increase in COVID-19 incidence within the second pandemic wave led once again to country-wide school closures on December 18, 2020. From November to December 2020 the multicentre intervention study “Bundesweites Forschungsnetz “Angewandte Surveillance und Testung” (B-FAST)” was initiated with the intention of developing comprehensive and scalable surveillance strategies which can be performed autonomously in schools, to gain school-based epidemiological data and to find concepts to keep educational facilities open safely. The study exploited RT-qPCR-based pooled testing, in which samples were grouped (pooled) instead of testing individual swabs from every single study participant. This approach naturally led to a cluster randomized trial using classes as units of randomisation. Objectives of the study were to evaluate acceptance, feasibility and efficacy of age-adapted swab sampling methods for pooled RT-qPCR for the detection of SARS-CoV-2.

## 2 Methods

### 2.1 Study sites and recruitment

This prospective multicentre study was performed between November 9 and December 23, 2020 in 14 primary and secondary schools. Sites were selected from volunteering schools in five communities in Germany (Cologne, Duesseldorf, Rhein-Neckar-county near Heidelberg, Homburg/Saar, Munich). School selection aimed to cover a variety of population densities and social settings, not to be population-representative. Site recruitment required approval of communities, school boards and local health authorities. After recruitment, all students and staff attending one of the schools were offered participation in the study. National COVID-19 preventive measures applied in schools during the study period are described in the Supplement.

The study was approved by the ethics committee of the medical faculty, University of Cologne (registration number 20–1463) and the respective ethics committees of all participating study sites.

### 2.2 Participants

Participants in the study were students attending the participating schools. Additionally, school staff was invited to participate. School staff comprised all employees at the school with direct contact to students such as teachers, secretaries, janitors, caregivers and kitchen staff. Depending on their age, students and/or their parents/guardians as well as participating staff were required to give written informed consent based on a wide range of information. A written invitation and description of the study for parents and an age-adjusted version for students was distributed. The study team visited each class to introduce themselves and to demonstrate applied methods. An age-adjusted explanatory video was presented. A videoconference for school staff and parents at each school was realised. A hotline for further questions was implemented. After receiving all information, all legal guardians and participants (aged 8 years and above) gave written consent. Participation was voluntary and consent could be withdrawn at any time.

### 2.3 Randomisation

**Fig. 1:**
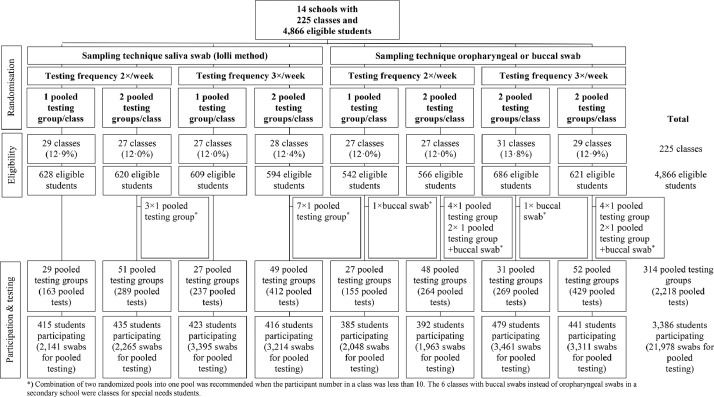
CONSORT chart for students by randomised groups. Students were randomised into the three factors sampling technique (saliva swabs using the ‘lolli method’ vs. oropharyngeal or buccal swabs), testing frequency (two vs. three times per week), and number of pooled tests per class (one pooled test containing all swabs of a class vs. two pooled tests each containing half of the swabs taken in the respective class) using a 2 × 2 × 2 incomplete block design. See Fig. 4b for staff participation and testing.

Randomisation of students was performed in clusters with classes serving as units. Three factors were randomised: sampling technique (saliva swabs using the ‘lolli method’ vs. oropharyngeal or buccal swabs), testing frequency (two vs. three times per week), and number of pooled tests per class (one pooled test containing all swabs of a class vs. two pooled tests each containing half of the swabs taken in the respective class) using a 2 × 2 × 2 incomplete block design with a 1:1 allocation ratio for each factor (CONSORT flow diagram displayed in Fig. 1). Random assignment was done centrally, stratified by school, blocked with length 8 and based on pseudo-random numbers generated with Excel 2019 (Microsoft Corp., Redmond, WA, USA) by a statistician (MH). Randomisation results were provided to local personnel that communicated these to study participants only after obtaining informed consent.

### 2.4 Procedures

Specimen collection and laboratory procedures are described in the Supplementary appendix.

### 2.5 Outcomes

The designated primary study outcome criterion was overall acceptance of surveillance methods on the individual level. It was measured by participation rate, i.e. the number of students with informed consent divided by the number of eligible students as well as dropout rate, i.e. the number of students discontinuing the study prematurely divided by the number of students with informed consent. Differences in these two rates between randomised testing strategies were a secondary outcome criterion. Other secondary outcomes include the number of positive pooled SARS-CoV-2 RT-qPCR tests and the number of SARS-CoV-2 RNA positive individuals detected in groups with positive pooled tests.

### 2.6 Sample size

Assuming, (1) a proportion of 50% overall acceptance (consent or no cancellation) with oropharyngeal/buccal swab sampling, (2) 75% with saliva swab sampling, (3) average group size of *n* = 20 with a coefficient of variation of 20%, (4) intra-class correlation (ICC) of 0·1 [21], (5) power of 80%, (6) two-sided type I error 5%, then *n* = 2 × 9 classes with a total of approx. *n* = 360 students with allocation ratio 1:1 are sufficient for the 25% (target) difference in the respective proportions to be detected with 80% probability (Stata/SE 16·1, StataCorp LLC, College Station, TX, USA). The comparisons with regard to the number of pooled tests per class and frequency of testing were aimed to demonstrate non-inferiority (with a margin of 5%). For relevant subgroups *n* = 180 students per comparison group are sufficient to estimate 95% confidence intervals (CI) around rates with a half-width of at most 18 percentage points (worst case at a rate of 0·5 using a design effect of 2·9 to account for the clustered nature of the data). Moreover, a total of *n* = 5000 (10,000) students yield a half-width of approx. 5% (3%).

### 2.7 Statistical analysis

Categorical data were summarized by absolute and relative frequencies (incidence rate as per 100,000 persons and time period), continuous data by median and range. Odds ratios with 95% confidence intervals were derived based on generalised linear mixed models with random intercept for school and class within school. The random intercept for school was omitted if small numbers did not allow for model estimation. In these cases, results were compared against an exact logistic regression for plausibility. ICCs were calculated from random intercept logistic models [22]. Analyzes are based on the intention-to-treat principle for randomized comparisons. *P* values < 0·05 were considered statistically significant without adjusting for multiple testing. Community-based incidences were queried from the Robert-Koch-Institute [23], the local health authorities and the virology departments involved. Statistical calculations were done using SAS software (version 9·4, SAS Corp., Cary, NC, USA). The B-FAST study was registered at the WHO International Clinical Trials Registry Platform (ICTRP) via the German Clinical Trials register (registration number: DRKS00023911).

### 2.8 Role of the funding source

The funders had no role in study design, data collection and analysis, decision to publish, or preparation of the manuscript. The study team (ZCC, JS, AK, IS, AJ, FD, HH, MH, BH, KJS, JR, JD) had access to the underlying data. The corresponding authors had full access to all the data in the study and had final responsibility for the decision to submit for publication

## 3 Results

### 3.1 SARS-CoV-2 incidence at study sites and demographic data

**Fig. 2:**
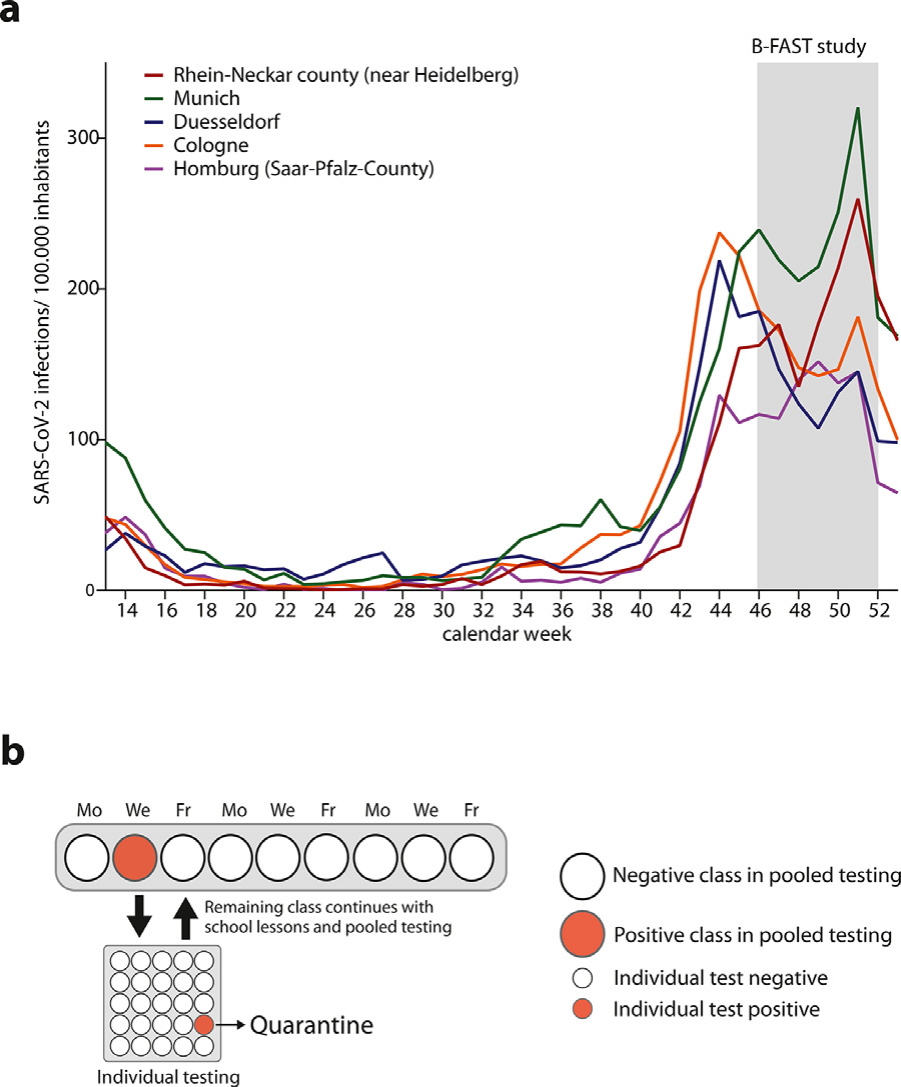
SARS-CoV-2 incidence at study sites and study design. a. Number of SARS-CoV-2 infections/100,000 inhabitants in the participating cities/counties from calendar week 14–53 in 2020. The B-FAST study period is highlighted in grey. b. B-FAST study design in which swabs were taken from an entire class several times per week (Monday, Wednesday and Friday (shown) *or* Monday and Thursday). Swabs were pooled and tested by RT-qPCR. Detection of a positive pooled test (red dot) triggered individual testing of the entire class by RT-qPCR and subsequent identification of a single or several positive individuals who were quarantined. The remaining students continued with school lessons and scheduled pooled testing (For interpretation of the references to color in this figure legend, the reader is referred to the web version of this article.).

This multicentre study was performed between November 9 and December 23, 2020 in five German cities/counties. The study cites were simultaneously affected by the second COVID-19 wave with fairly comparable SARS-CoV-2 incidence rates (Fig. 2a). Using a longitudinal study design, swabs were taken from entire school classes two or three times per week for at least three consecutive weeks (Fig. 2b). Detection of a positive pooled test triggered individual re-testing of the entire class or group by RT-qPCR and subsequent identification of a single or several positive individuals which were quarantined (Fig. 2b). The remaining class or group members continued with school lessons and scheduled pooled testing.

In this study, 3386 students from 225 classes and 584 staff members in 14 schools participated (Fig. 1). The number of participating students per school varied by a factor of 20 from a small primary school contributing 33 children to 586 participants in the second-largest secondary school (Supplementary Table S1). The median class size was 22 students for primary and 23 students for secondary schools (Supplementary Table S2). Demographic data are provided in Supplementary Table S2. Median age of students was 12 years (range 5–21) with about half of the students being female (*n* = 1708, 51·9%). Median age of school staff was 44 years (range 16–82) and they were mostly female (415, 71·6%) (Supplementary Table S2).

### 3.2 Acceptance, dropout rates and feasibility of applied methods

When performing repetitive testing with potentially painful or uncomfortable sampling methods in students, acceptance of and adherence to the procedure is an important aspect to consider. Exploiting the cluster randomised trial design, we first determined acceptance and dropout rates of different sampling modalities for pooled RT-qPCR. Saliva swab samples obtained by sucking on a test swab for 15 s (called ‘lolli method’) were compared to standard oropharyngeal swabs in secondary schools and buccal swabs in primary schools. In addition, acceptance and feasibility of testing frequencies (2 ×/week vs 3 ×/week) as well as the number of pooled tests per class (entire class in one pooled test vs splitting of the class in two pooled tests) were determined. Randomisation of classes into evenly distributed strata evaluating the three different factors is shown in Fig. 1. During the study period, a total of 21,978 swabs were taken and combined in 2218 pooled RT-qPCR analyzes (Fig. 1). The median number of swabs pooled for RT-qPCR analysis was 10 (range 1–26) and, per randomized group the median was 15 for full classes and 8 for split classes, respectively (Supplementary Table S4).

Overall acceptance as measured by participation rate was 69·6% for students (3386 of 4866 students who were offered participation). The groups in the 3 randomised comparisons had participation rates between 68·9 and 70·3% with no statistically significant differences between randomised groups (Table 1). Per school, the rate varied between 45·6 and 86·8%. However, only three schools had a participation rate below 66% meaning that at least two thirds of all students were participating in 11 schools (Supplementary Table S1). Delving into the class level, participation rates ranged between 3·3% (1 in 30) and all children of a single class (Supplementary Table S3). Communication of grouping results to study participants had no immediate impact on participation rates in the different groups (data not shown).

**Table 1:** Acceptance of different sampling techniques performed with students as measured by participation and dropout rate.

	Sampling technique		Pooled testing frequency		Number of pooled tests per class		Total
	Saliva swab (lolli method)	Oropharyngeal swab (secondary schools) or buccal swab (primary schools)	2 × /week	3 × /week	1 pooled test	2 pooled tests	
**Participation rate**	1689/2451 (68·9%)	1697/2415 (70·3%)	1627/2356 (69·1%)	1759/2510 (70·1%)	1702/2465 (69·0%)	1684/2401 (70·1%)	3386/4866 (69·6%)
Model based*95% CI for odds ratio, p value	0·93 (0·74–1·17), *p* = 0·8		0·97 (0·77–1·22), *p* = 0·5		0·99 (0·79–1·24), *p* = 0·9		
**Dropout rate (% from participating)**	4/1689 (0·2%)	14/1697 (0·8%)	12/1627 (0·7%)	6/1759 (0·3%)	7/1702 (0·4%)	11/1684 (0·7%)	18/3386 (0·5%)
**Model based**†**95% CI for odds ratio, p value**	0·27 (0·09–0·94), *p* = 0·04		2·26 (0·75–6·13), *p* = 0·15		0·66 (0·24–1·84), *p* = 0·43		

CI, confidence interval; ICC, intra-class correlation.

⁎ Estimates from common mixed logistic model with random intercept for school and class (ICC 0·20), and sampling technique, weekly frequency and number of pools per class as fixed effects.

† Similar model as for participation rate omitting the school intercept (ICC for classes only 0·89) because the small number of dropouts did not allow to include it.

Throughout the 3-week study period, the overall dropout rate was low with only 18 of 3386 participating students (0·5%) terminating the study prematurely (Table 1). Students sampled by oropharyngeal or buccal swab were statistical significantly more likely to end the study prematurely than students who were tested by saliva swab sampling with 4 (0·2%) vs 14 students (0·8%) dropping out, resulting in an odds ratio of 2·26 (95% CI 0·09 to 0·94; *p* = 0·04, controlled for weekly testing frequency and number of pooled tests per class). The two other comparisons (testing frequency and number of pooled tests per class) showed no statistically significant difference regarding dropout rates (Table 1). All schools and classes completed the three-week study period as scheduled except for two schools in which testing had to be terminated prematurely after the first visit in the third week because of nationwide school closures (Supplementary Table S4). Eleven of 225 classes continued testing into week 4 or 5 to follow-up on putative transmissions after detection of SARS-CoV-2 positive individuals in week 2 or 3 of the study (Supplementary Table S4). Of note, for re-testing of individual children by RT-qPCR, 487 of the 493 expected samples (98·8%) could be collected (not shown), indicating again high acceptance of the applied procedure in both students and parents.

Applied methods were feasible with expected turnaround times for transport and laboratory diagnostics. Pooled RT-qPCR test results were communicated during the day of sampling in all cases. For groups with higher numbers of pooled tests due to splitting of classes into two test groups, a total number of 1394 RT-qPCR reactions had to be performed. In contrast, 824 RT-qPCRs were performed in non-split groups (Fig. 1). Higher RT-qPCR workload had no negative impact on timely communication of test results.

### 3.3 Test results for students

**Fig. 3:**
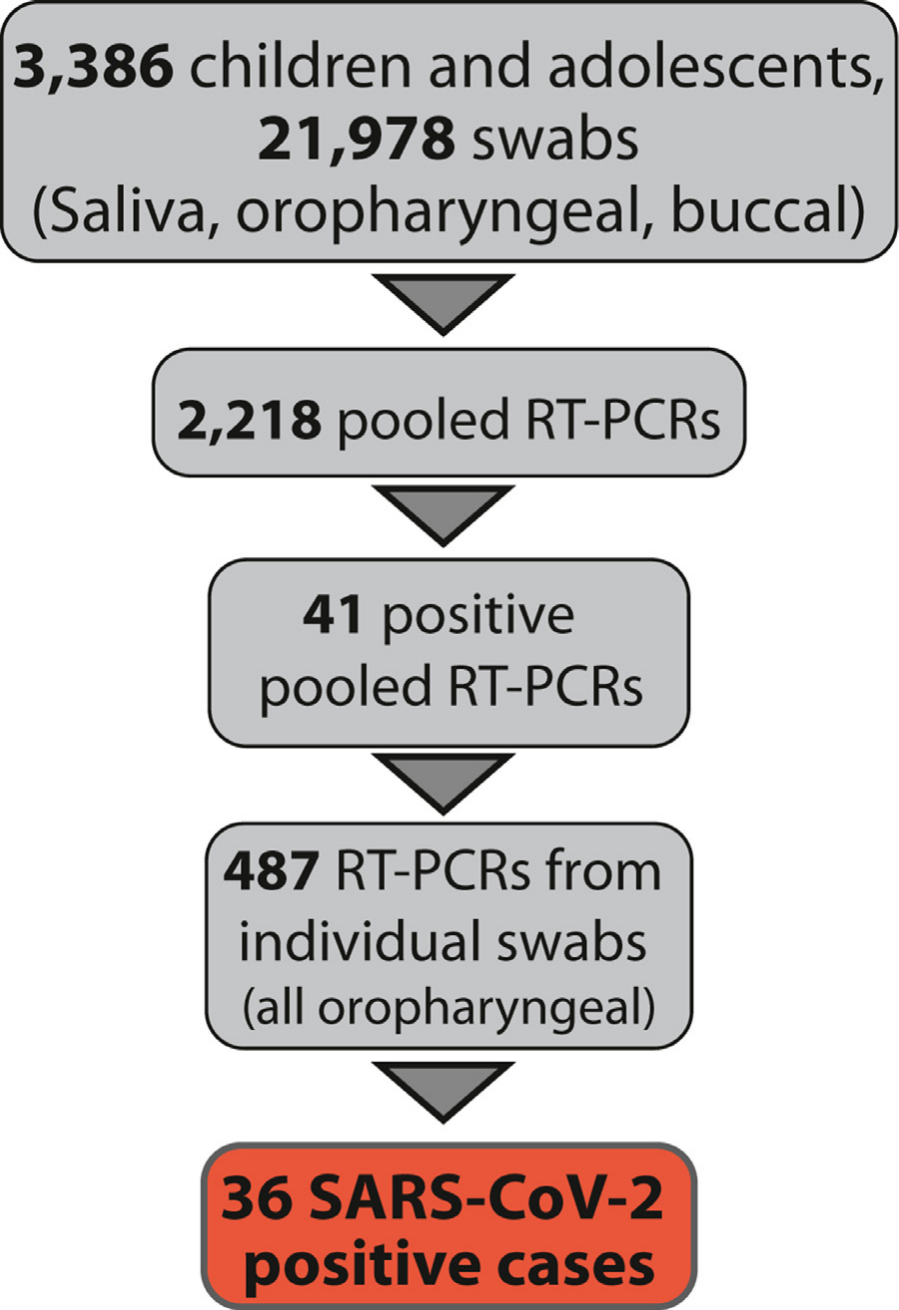
Overall study results for students. A total of 21,978 swabs were taken and combined in 2218 pooled RT-qPCR tests. We detected 41 positive pooled tests leading to 36 SARS-CoV-2 cases which could be identified by individual re-testing.

In 2218 pooled RT-qPCR analyzes from 21,978 swabs we identified 41 positive pooled tests among students (1.8% of pooled analyzes) of which 36 SARS-CoV-2 positive cases were identified by individual re-testing (Fig. 3). The rate of classes with SARS-CoV-2 positive cases was 12·4% (28 of 225 classes), with 4 out of 8 primary schools (50%) and 5 out of 6 secondary schools (83·3%) being affected (Supplementary Table S5). 36 SARS-CoV-2 positive individuals were detected by individual re-testing of the entire pooled group as scheduled according to the study protocol (Fig. 3). The majority of SARS-CoV-2 positive pooled tests led to detection of a single SARS-CoV-2 positive individual (*n* = 29). Two pooled tests led to identification of two cases per pooled test and one single positive pooled test led to identification of three cases (not shown). The failure of correlating positive pooled tests to positive individuals in 5 cases may be explained by low viral loads in the affected individuals which could have led to negative results upon re-testing using oropharyngeal swabs the next day. RT-qPCR cycle threshold (Ct) values of pooled RT-qPCRs are provided in Supplementary Fig. S1. The five pooled samples that failed to detect positive individuals showed Ct-values ≥ 38 indicating low viral loads (Supplementary Fig. S1). Of note, repetitive testing two or three times per week ensured continuous surveillance of classes presenting with these discrepant test results.

**Fig. 4:**
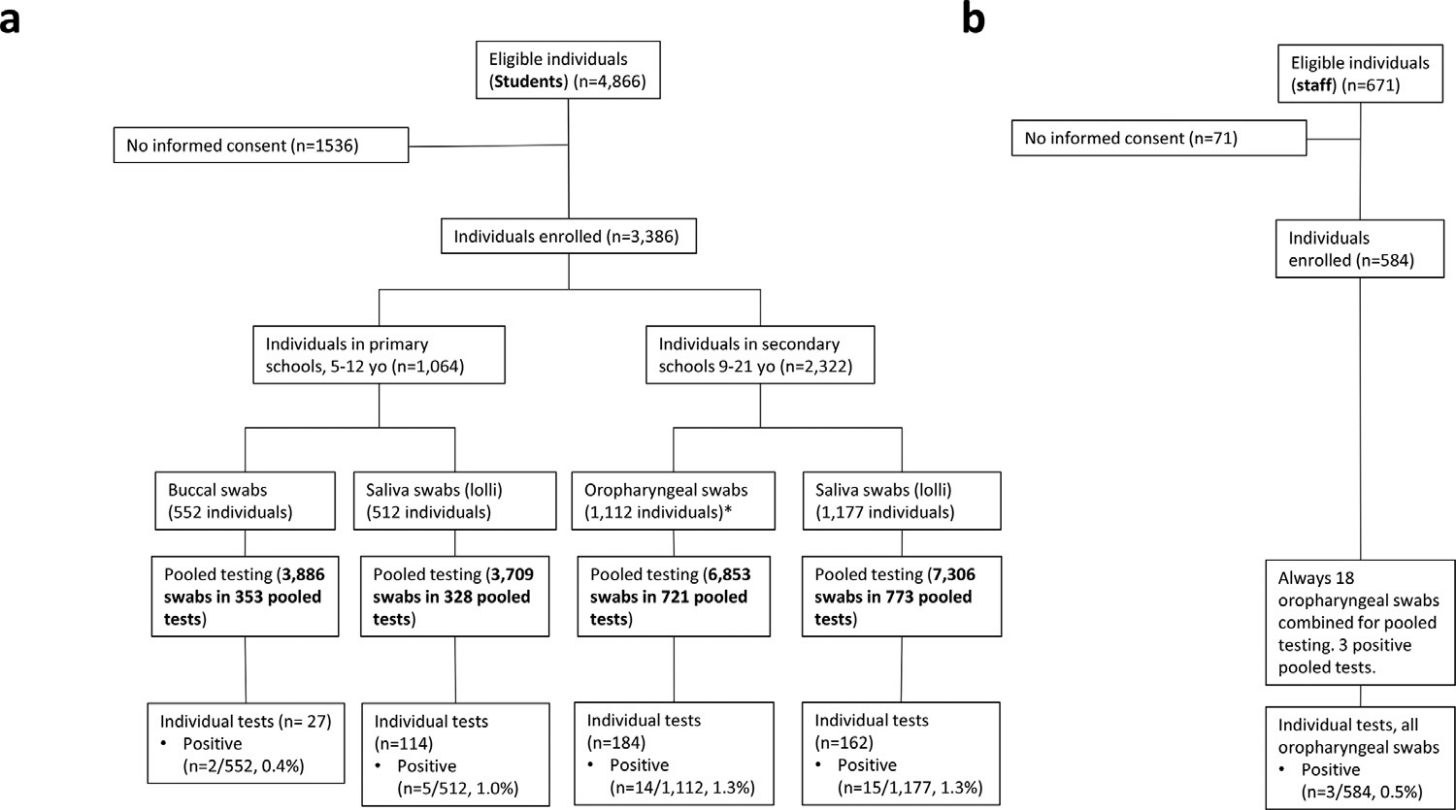
Selected study results for sampling method in students and staff. a. Flow diagram showing selected study results for one of three randomised factors which tested for three age-adjusted swab sampling methods (oropharyngeal swabs, buccal swabs and saliva swabs (lolli method). Number of eligible and enroled students and study results for pooled and individual RT-qPCR testing. (yo: years old). For CONSORT flow diagram of the entire study see Fig. 1. b. Number of eligible and enroled staff members and study results for pooled and individual RT-qPCR testing.

**Fig. 5:**
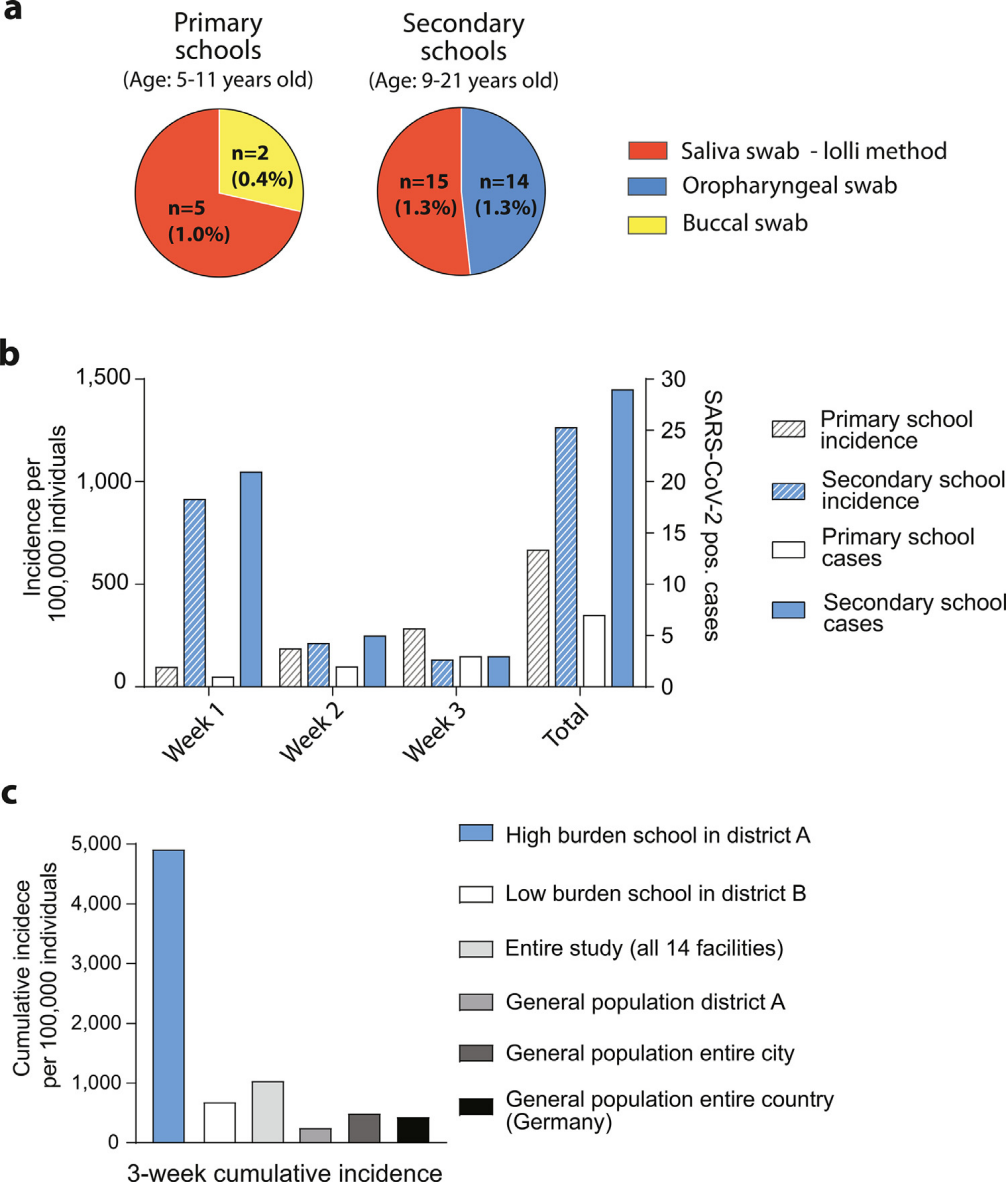
Positivity rate dependent on sampling method and incidence rates. a. Rate of positive students identified with different sampling methods used for pooled testing in total numbers and percentage. Lolli method: red pie; buccal swab: yellow pie; oropharyngeal swab: blue pie. b. Number of SARS-CoV-2 positive students (right Y-axis) and incidence rate (left Y-axis, per 100,000 individuals) per week in secondary and primary schools. Note that week 1 contains asymptomatic prevalent individuals many of whom were identified on the first day of testing. Secondary schools: blue bars; primary schools white bars. Incidence rate: hatched bars; cases: open bars. c. Cumulative incidence (per 100,000; three-week period) identified in a high burden school situated in a city district with lower socioeconomic status (secondary school 4, blue bar), a low burden school (secondary school 5, white bar), in the entire study (all 14 schools, light grey bar), the general population of the respective Cologne district of secondary school 4 (medium grey bar), the general population of the city of Cologne (dark grey bar) and the entire country (Germany, black bar) (For interpretation of the references to color in this figure legend, the reader is referred to the web version of this article.).

The relatively high acceptance of applied procedures and low dropout rates in all randomised groups allowed us to also explore secondary endpoints such as the effectiveness of different sampling techniques with regard to detection of SARS-CoV-2 positive cases. In secondary schools, we compared pooled testing of standard oropharyngeal swabs (7306 swabs) to saliva swab samples (lolli method) (6853 swabs) (Fig. 4a). There was no statistically significant difference in the number of SARS-CoV-2 positive students identified from pooled saliva samples using the ‘lolli method’ compared to those identified from pooled oropharyngeal swabs (15 vs. 14 cases; 1·3% vs. 1·3%; OR 1·1; 95%-CI 0·5–2·5), (Fig. 5a, and Supplementary Table S5). In primary schools, the ‘lolli method’ (3709 swabs) detected more cases than buccal swab (3886 swabs) sampling (5 vs. 2 cases; 1% vs 0·4%; OR 2·7; 95%-CI 0·5–15·5), (Figs. 4a, 5a and Supplementary Table S5).

Using the longitudinal study design we next assessed 7-day incidence rates in students over time. In secondary schools, the incidence was high during the first week (916/100,000) with 11 of 29 (37·9%) prevalent individuals detected on the first day of testing (Fig. 5b, Supplementary Table S5). In week 2 and 3, the incidence and case numbers declined sharply. A similar effect was not observed in primary schools where overall case numbers were low. Based on all sampling methods, the cumulative 3-week incidence for primary schools was 564/100,000 (6/1064, additionally 1 infection detected in week 4) and 1249/100,000 (29/2322) for secondary schools (Fig. 5b).

The majority of SARS-CoV-2 positive students identified in the entire study were detected in a single secondary school situated in the city of Cologne. This school contributed 17 of 36 cases (47%) (Fig. 5c). The affected school is a medium sized junior high school with 758 students and 74 employees. Classes with positive pooled tests were evenly distributed throughout the six grades with detection of mostly one SARS-CoV-2 positive case per class. However, one class in grade ten had four SARS-CoV-2 positive individuals identified with two pooled tests (see also phylogenetic strain analysis Supplementary Fig. S2 and Supplementary Table S8).

The 3-week cumulative incidence for this high-burden school was 10-fold higher than the incidence of the general population of the city of Cologne (4913 vs 463 cases/100,000 individuals) and almost 20-fold higher than the incidence of the population living in the respective city district (250 cases/100,000 individuals) during the study period, indicating many asymptomatic and/or undetected cases in this community (Fig. 5c). We performed a structured interview evaluating symptoms and additional infections among family members. Results are provided in the supplementary appendix (‘detailed analysis of a high burden school’).

There is growing evidence that socio-economic inequalities are important factors influencing SARS-CoV-2 incidence and hospitalisation rates [24]. To identify possible reasons for such high numbers of SARS-CoV-2 positive students, we compared socio-economic factors of the respective city district (district A) to factors of a second Cologne city district (district B) with a secondary school showing a much lower number of SARS-CoV-2 positive students (Fig. 5c). Results are provided in the supplementary appendix (‘detailed analysis of a high burden school’).

### 3.4 Test results for staff

Staff participation was high with nearly 85·3% of staff taking part in the study (584 of 671 eligible individuals, Fig. 4b, Supplementary Table S5). We identified three positive pooled RT-qPCR tests among staff members resulting in three SARS-CoV-2 positive cases upon individual re-testing. All SARS-CoV-2 positive staff members were teachers. The 7-day-incidence in this group was 171/100,000 individuals.

The three SARS-CoV-2 positive staff members were employed at three different schools (Supplementary Table S6). In the follow-up, no classes that were taught by the infected teachers showed positive pooled test results.

### 3.5 Putative in-class transmissions and phylogenetic analysis

By performing sequential testing of individual classes over time, we found that 6 of 28 classes with infections (21%) encountered new cases after detection of the first SARS-CoV-2 positive case (Supplementary Tables S5 and S8). Three classes in two primary schools and three classes in two secondary schools were affected. The last pooled RT-qPCR testing conducted was negative in all classes.

A phylogenetic network analysis was performed with genomes of SARS-CoV-2 strains isolated from six students attending three classes with putative onward transmission events (Supplementary Fig. S2, Table S8). Additional genome sequences from five SARS-CoV-2 strains isolated in the high-burden school described above were added to the analysis. There were two genetically identical strains detected in siblings attending different classes. All other strains were genetically distant. This was also the case for strains isolated from individuals involved in putative in-class transmission events indicating that transmission did not occur in the school environment.

## 4 Discussion

In this prospective multicentre study, we demonstrate that pooled RT-qPCR-testing of children and adolescents is feasible and can therefore serve as an appropriate tool for surveillance of SARS-CoV-2 infections in schools. Our data suggest that saliva swab samples collected with the ‘lolli method’ are suitable for SARS-CoV-2 surveillance in this setting. Thus, combining both methods results in a simple and fast-to-perform as well as largely scalable test strategy. Acceptance of sampling procedures is key to successful surveillance strategies in children and adolescents. Although overall dropout rates were low in our study, there were statistical significantly less study discontinuations when sampling was performed using the ‘lolli method’ compared to standard oropharyngeal or buccal swabs. This should be an important point to consider when repetitive testing for SARS-CoV-2 in schools is planned for longer periods e.g. several weeks or months.

In our study the samples had to be taken by trained medical staff due to regulatory reasons. However, children and adolescents can easily take saliva swabs with the ‘lolli method’ themselves, without putting others at risk of transmission. Compared to a strategy of self-testing at home, our approach has also the advantage of better control over the quality of the test procedure and the adherence to it. Successful SARS-CoV-2 mass testing requires high participation rates and long-term compliance to maximise potential benefits. Testing with LFD or rapid antigen-tests in classrooms appears to be much more time consuming and necessitates more teacher involvement. The higher sensitivity and specificity are further advantages of RT-qPCR-testing [18]. In addition, pooled testing can also be used to increase surveillance capabilities in resource-limited settings [25].

This study was performed from November to December 2020, when a second wave of the SARS-CoV-2 pandemic caused constantly increasing incidence rates in Germany and ultimately led to the closure of schools in late December (Fig. 2a). In this evolving epidemiological situation, we were able to detect 36 SARS-CoV-2 positive cases among students. Three-week incidence rates in older students (1249/100,000 in secondary schools) were approximately two-fold higher than those identified in younger children (564/100,000 in primary schools). This is in line with the observation of others showing higher infection rates in teenagers than in younger children [10,[26], [27], [28]–29]. However, it is important to note that many cases were detected in a single secondary school making this comparison less reliable. In addition, half of the children visiting primary schools were tested using buccal swabs (*N* = 552) which led to detection of fewer cases than saliva swab sampling with the lolli method. Although our sample size was not large enough to compare buccal swab and saliva swab sampling in a statistically reliable manner, it is conceivable that buccal swab sampling missed infections in younger children.

The majority of cases were detected in the first of three study weeks, and all infections were diagnosed in individuals coming to their respective facility without reporting symptoms to their teachers or other school personnel. This underlines the potential of our approach to identify asymptomatic or pre-symptomatic persons, and early quarantining of these individuals may prevent larger outbreaks in the affected facilities. We found six potential onward transmissions in 28 school classes with positive cases. In three cases, phylogenetic analyzes of viral strains revealed that in-class transmission was an unlikely event. The low number of sequential infections within classes may indicate that transmissions in classrooms are rare which is in-line with cluster detection studies based on seropositivity of children visiting the same class [30,31]. However, we cannot exclude transmissions among individuals of different classes, which may have been acquired outside of the school environment. In addition, rapid quarantining of pre-symptomatic children as performed in our study may have prevented in-class or in-school transmission. Uncertainty remains as to whether the spread of new variants of SARS-CoV-2 may increase the risk for in-class transmission.

SARS-CoV-2 infections detected in this study clustered in one secondary school resulting in 17 out of 36 individual cases. This finding demonstrates a large heterogeneity of incidence rates depending on regional and social indicators, which should be considered when estimating prevalence rates in children and adolescents based on surveillance studies performed in educational settings. Intriguingly, the 3-week cumulative incidence of this high-burden school was almost 20-fold higher than the incidence rate calculated based on registered cases of the general population living in the respective city district. This suggests a large discrepancy between the number of confirmed cases and true infections in this area. A possible reason for the high incidence rate identified in this school may be found in socio-economic disparities of families living in the respective city district. Recent data emphasise the higher rate of SARS-CoV-2 infections in migrant communities and populations with challenging socio-economic conditions [24,32]. We made similar observations when comparing two schools and their respective city districts with distinct socio-economic backgrounds (Supplementary Table S7). Additional investigations are required to confirm these preliminary findings on socio-economic factors influencing SARS-CoV-2 incidence rates in schools. Recent epidemiological data highlight the strong association of incidence rates in educational settings with those found in nearby communities or regions [14]. It is conceivable that systematic SARS-CoV-2 testing in educational settings could function as a sentinel surveillance tool to better estimate true incidence rates in certain districts and environments.

Our study has several limitations. Since students participated on a voluntary basis, we could not include all individuals in each class. Difficulties in understanding the purpose and the conduct of the study due to language barriers with parents may have contributed to the relatively low rates of participation in some schools, and we therefore may have missed SARS-CoV-2 infections or transmission events. In line with this assumption, we found that the school with the highest number of SARS-CoV-2 positive cases had one of the lowest participation rates (45·6%, Supplementary Table S1). In individuals with a positive test result we were only partially able to trace potential infection routes outside schools, e.g. within families and household contacts which was due to regulatory reasons.

Participating schools were chosen by city and district administration boards and selection aimed to cover a variety of population densities and social settings. However, the sample was not population-representative and socio-economic comparisons between participating or non-participating schools could not be made for all schools and districts.

Despite a relatively large sample size, no predictions can be made regarding the validity or sensitivity of the applied sampling methods. In addition, nasopharyngeal swabs could not be performed as a screening measure in children due to regulatory reasons. For this reason, we performed a second study in parallel to the multicentre study described here, which evaluated the SARS-CoV-2 detection rate of RT-qPCR tests performed with saliva swab samples (lolli method), (Dewald et al., under review). This study shows a good detection rate of 91% for saliva swab samples collected with the ‘lolli method’ when viral loads of the corresponding naso-/oropharyngeal swabs are between 10³ and 10^6^ copies/mL. Of note, SARS-CoV-2 LFD or rapid antigen tests are known to show a significantly lower detection rate under similar conditions [18,33,34].

In conclusion, we describe a simple and feasible method using pooled RT-qPCR-tests of saliva swab samples for SARS-CoV-2 surveillance of schools. This method is suitable for autonomous application and represents a potential important tool to detect and limit SARS-CoV-2 infections in educational facilities. Autonomous application of our approach is currently being explored as the primary SARS-CoV-2 surveillance tool in primary schools across Germany.

## Data sharing statement

Deidentified data will be made available in the context of the German network for academic research on COVID-19 (https://www.netzwerk-universitaetsmedizin.de/).

## Contributors

All main authors contributed to the conception and design of this study, the acquisition, analysis and interpretation of data, drafting the manuscript and revising it critically for important intellectual content, gave approval to the final version to be published and agreed to be accountable for all aspects of the work in ensuring that questions related to the accuracy or integrity of any part of the study were appropriately investigated and resolved. All authors contributed to all sections relevant to their experience and helped finalize the text and content.

The B-FAST Study Group consists of scientists and partners, who contributed important work to the study as they performed the tests in schools (ZCC, JS, AK, RS, HL, LS, MT), developed and performed the pooling technique (EH, GHR, EK, RK, SS, NS, TP, SL, AKS, MAB, JT, BM, KB, AMH, UVB, AK), served as scientific advisors (CH, MW, GH), managed the administrative framework (SGF, EK, SM, NG, CP, JW), performed statistical analysis (MH, KSH) and partnered as public health scientists (AK, RB, AW).

## Declaration of Competing Interest

All authors declare no competing interests.

## Appendix Supplementary materials

Image, application 1
