# Supplementary material for: Pooled RT-qPCR testing for SARS-CoV-2 surveillance in schools - a cluster randomised trial
Source: eClinicalMedicine. 2021 Aug 25;39:101082. doi: 10.1016/j.eclinm.2021.101082 (PMC8384501; doi:10.1016/j.eclinm.2021.101082)
Supplement: Supplementary file 1 [file mmc1.docx]

**Supplementary appendix for:**

**Pooled RT-qPCR testing for SARS-CoV-2 surveillance in schools**

**- A cluster randomised trial**

This document includes:

1. Supplemental methods
2. Supplemental results
3. Supplemental figures S1-S3.
4. Supplemental tables S1-S9
5. Names and affiliations of the B-FAST study group
6. References supplementary appendix

**Supplementary methods**

**National COVID-19 preventive measures in schools during study period**

In primary schools, face masks for staff were mandatory at all times; face masks for children were mandatory when not seated at their desks. Hand sanitation stations installed in hallway at the school entrance were available in 75% of facilities. In secondary schools, face masks for staff and students were mandatory at all times. Hand sanitation stations installed in hallway at the school entrance were available in 83% of facilities. Classes were not split to reduce the number of students per classroom in any of the participating schools. There was no distance learning applied in any of the participating facilities.

**Standard care for testing and quarantine during study period**

During the study period, there was no standard test programme for schools applied. Students were attending school in full class size under preventive measures as described above. Students were only tested outside of schools when meeting criteria for testing defined by health authorities. Criteria for testing included typical symptoms (fever, coughing, loss of gustatory and/or olfactory sense), close contact to SARS-CoV-2-positive case and return from high incidence region.

SARS-CoV-2 positive persons were quarantined in home isolation for 14 days. Contact tracing was performed by health authorities and close contacts (category 1 - CP1) were quarantined as well. Testing of close contacts was only performed if contacts showed symptoms. No negative test was mandatory when terminating quarantine. How many students of one class were identified as CP1 depended on several factors (length of contact, wearing of masks, venting of class room, etc).

The B-FAST study offered a standardised test programme and deviant quarantine measures (CP1 were allowed to attend school, see “Quarantine”) to ensure a safe schooling for as many students as possible.

**Sequencing of positive samples**

For sequencing of SARS-CoV-2 positive samples at the Cologne site, 500 µl of swab media were used to purify nucleic acids using the MagNa Pure 96 automatic nucleic acid extraction instrument and the Viral NA large volume Kit. At the Duesseldorf site, the EZ1 Virus mini kit v2.0 (Qiagen) was used. Sequencing was performed according to COVID-19 ARTIC v3 ^1^. Briefly, cDNA was synthesized with random primers. Amplification of the SARS-CoV-2 full genome was achieved with 219 primers split into two primer pools (123 primers in pool 1 and 96 in pool 2). These amplicons were then used to construct the library for sequencing.

Sequence quality was assessed with the Nextclade tool (https://clades.nextstrain.org/). Sequences containing >1000 missing sites (Ns) and/or >10 mixed sites were considered to miss too much data and discarded.

Phylogenetic analyses were performed using the Mega X software^2^. Multiple alignments of DNA sequences were generated using the Muscle tool using UPGMB for clustering with a minimum Diag. Length of 24 and maximal 16 iterations^3^. The pairwise genetic distance and phylogenetic reconstruction were carried out using maximum likelihood methods assuming a Tamura-Nei model of base substitution and with 100 bootstrap replications.

**Specimen collection for pooled testing**

Samples were collected with a standard dry swab (polystyrol sticks with viscose tip in single tube without medium, no intended breaking point). The study team (physician and trained medical students) entered the classroom and school activities were paused for 5-8 minutes.

For saliva sampling the sealed swabs were distributed to every participant. To this end, the participants placed the swab in their mouth for 15 seconds until the swab was soaked with saliva. Sucking on the swab was allowed (similar to a lolli). In the manuscript, the method is called ‘saliva swab’ and ‘lolli method’.

For oropharyngeal and buccal sampling participants were asked to open their mouth while a trained member of the study team performed the sampling procedure. In students attending secondary schools, an oropharyngeal (throat) swab was taken. In children attending primary schools, a buccal swab was performed. Nasopharyngeal swabs were not performed in this study. Swabs were inserted back in the tube and collected in a labelled polyethlene sample bag. No transport medium was used at this time and the swabs were transported as dry/native samples. Estimated transport times to local laboratories varied between 5 and 30 minutes, depending on location of the facility.

Swabs were taken from entire school classes twice or thrice per week. Detection of a positive pooled test led to individual testing of the respective pool participants by RT-qPCR (Manuscript Fig. 2b). The remaining students continued with school lessons and scheduled pooled testing.

Specimen collection for staff followed an adjusted protocol as described below.

**Pool processing and testing**

The samples derived from the different facilities were processed on the same day of collection. Up to 18 swabs of one protective bag, corresponding to one pooled test, were combined in 50 mL Falcon tubes pre-filled with 3 mL phosphate buffered saline (PBS) and vortexed for 30 seconds. In case of more than 18 swabs per pooled test, the samples were split in more than one 50 mL tube, in this case the first tube contained 4 mL PBS and the following tube contained no buffer. After a vortexing step of the first tube the PBS was transferred to the next tube which was vortexed again. This procedure was repeated until all tubes corresponding to one pooled test had been vortexed with PBS. After vortexing, 1 mL of PBS was used for SARS-CoV-2 RNA detection by quantitative reverse transciption-polymerase chain reaction (RT-qPCR), the remainder was stored as a back-up sample. The SARS-CoV-2 RNA detection was performed using validated kits for RT-qPCR on any of the following systems: Qiagen NeuMoDx, LightCycler 480 I&II, Cobas 6800, Alinity m, Hamilton, Nimbus, BioRad CFX96 Dx, according to the site availability. The samples derived from the different facilities were processed on the same day of collection as part of the routine diagnostic. Results were communicated to the study team on the same day.

**Specimen collection for individual testing**

In case of positive results in a pooled test sample in students, individuals of the respective pooled test were contacted and scheduled for individual testing on the same or the next day. Individual testing was performed in a dedicated room with a separate entrance and exit. Participants returned home after individual testing and waited for the results. Here, the sampling method was an oropharyngeal (throat) swab for all RT-qPCRs performed. For teachers and school staff, individual tests were performed using the backup samples.

**Processing of individual tests**

For dry swabs, each individual swab from the subjects of positive pooled tests was placed into a 5 mL tube, pre-filled with 2 mL PBS and vortexed for 30 seconds. 1 mL PBS was used to test for SARS-CoV-2 RNA and the remainder was stored.

For prefilled swab tubes, each individual swab from the subjects belonging to the positive pooled test was vortexed for 30 seconds. 1 mL of transport medium was used to test for SARS-CoV-2 RNA by RT-qPCR and the remainder was stored.

**Specimen collection for staff**

For school staff, a central testing room was implemented in the respective facility. Between 8 AM and 12 PM staff members were allowed to visit this room. This procedure guaranteed that all participants were able to attend specimen collection, even when starting work later in the day. Swabs of the first 18 staff members consulting the testing room were collected in the first collecting bag, swabs of staff members 19 to 36 were collected in the second bag and so on. The pool size was limited by size of the swabs and the volume of the 50 ml Falcon tubes.

Per sampling day two oropharyngeal (throat) swabs were taken. One was collected for pooled testing (18 swabs in one protective bag). The second one was labelled with an individual code and was stored at 4°C as a backup sample for individual testing in case of positive pooled tests. The reason for collecting a backup sample was to shorten the time required to receive individual test results in case of positive pooled tests among school staff members. This procedure avoided cancellation of lessons while waiting for individual retesting performed on the next day. By taking backup samples, same day results for school staff were guaranteed.

**Communication of test results and procedure thereafter**

Positive results of pooled tests were communicated to the study team on the same day. The study team contacted the respective school authorities and all persons who had been combined in the positive pooled test. Individual testing was performed on the same or the following day. Individuals that tested positive for SARS-CoV-2 RNA were contacted and reported to the local health authorities. If allowed by local health authorities, individuals that tested negative for SARS-CoV-2 RNA were allowed to attend their school the following days immediately after receiving the result (as long as they were study participants). Groups of a positive pooled test were followed-up for further SARS-CoV-2 infections for 2 weeks resulting in prolonged testing periods (up to 5 weeks in total, following the standard testing procedure described above) in groups with positive individuals identified in week 2 or week 3 of the study.

**Quarantine**

SARS-CoV-2-positive participants were quarantined at home for up to 14 days which was monitored by the local health authorities. Contact tracing was performed and individuals with increased risk of transmission, i.e. closest seated persons in the classroom, were identified designated as contact person category 1 (CP1). CP1 individuals without written informed consent for study participation were quarantined for 14 days following the standard procedures of health authorities. CP1 study participants were allowed to attend school under continuous pooled testing as described (if approved by local health authorities). If a positive pooled test did not result in one or more positive individual tests, no one was quarantined and pooled testing continued for two weeks.

**Data management**

Study data were collected and managed using REDCap (Research Electronic Data Capture) electronic data capture tools hosted by the Center for Clinical Studies (ZKS Köln), University Hospital of Cologne.

**Structured telephone interview with participants from the high burden school tested positive for SARS-CoV-2 during the study period**

Serving a content analysis, the interview was done by JS, a member of the B-FAST study team (MD, female, trained in patient communication). The caregivers of the participants that tested positive were contacted via telephone after the study period. Caregivers were familiar with the interviewer and the procedure as JS was part of the study team and the interview was announced in a newsletter after ethics committee approval. There was no pilot testing and no repeated interview. A catalogue of 4 questions was asked:

- Did the participant show symptoms during the SARS-CoV-2 infection (2 weeks before to 2 weeks after positive test)?
- What symptoms did the participant show (suggestions: coughing, common cold, sore throat, headache, melalgia, fatigue)?
- When was the onset of symptoms (days before/after positive test result)?
- Is there information about the infection chain (positive results within the family, friends, school, sport or other contacts)?

The duration of the interview was planned to be 5-10 minutes. Data was collected and managed at the University Hospital Cologne using Microsoft Excel.

**Supplementary results:**

**Detailed analysis of a high burden school**

The majority of SARS-CoV-2 positive students identified in the entire study were detected in a single secondary school situated in the city of Cologne. This school contributed 17 of 36 cases (47%) (Manuscript Fig. 5c).

A structured interview evaluating symptoms and additional infections among family members could be performed with 16 of 17 SARS-CoV-2 positive students and respective parents. Only two students reported symptoms prior to positive pooled testing (loss of gustatory sense and headaches). Eight students reported symptoms that developed after positive testing. Six students showed no symptoms at all. Five students reported another SARS-CoV-2 positive case in the same household, 11 cases did not know about other cases in their families or households.

To identify possible reasons for such high numbers of SARS-CoV-2 positive students, we compared socio-economic factors of the respective city district (district A) to factors of a second Cologne city district (district B) with a secondary school showing a much lower number of SARS-CoV-2 positive students (Manuscript Fig. 5c). District A clearly showed higher socio-economic disparities in all explored factors (e.g. educational status, rate of inhabitants with migrant background, unemployment rates, living space per inhabitant, details see Supplementary Table S7). To assure that city districts statistics match the situation within the investigated schools, we exemplary compared rates of individuals with migrant background and found similar results (in district A 72.4% for the city district and 75 % for students at the school, in district B 37.4% for the city district and 32.4% for the school).

**Supplementary Figures:**

**Fig. S1: RT-qPCR cycle threshold (Ct) values of pooled RT-qPCRs**

Green dots represent pooled tests that did not result in the detection of SARS-CoV-2 positive individuals upon retesting.

**
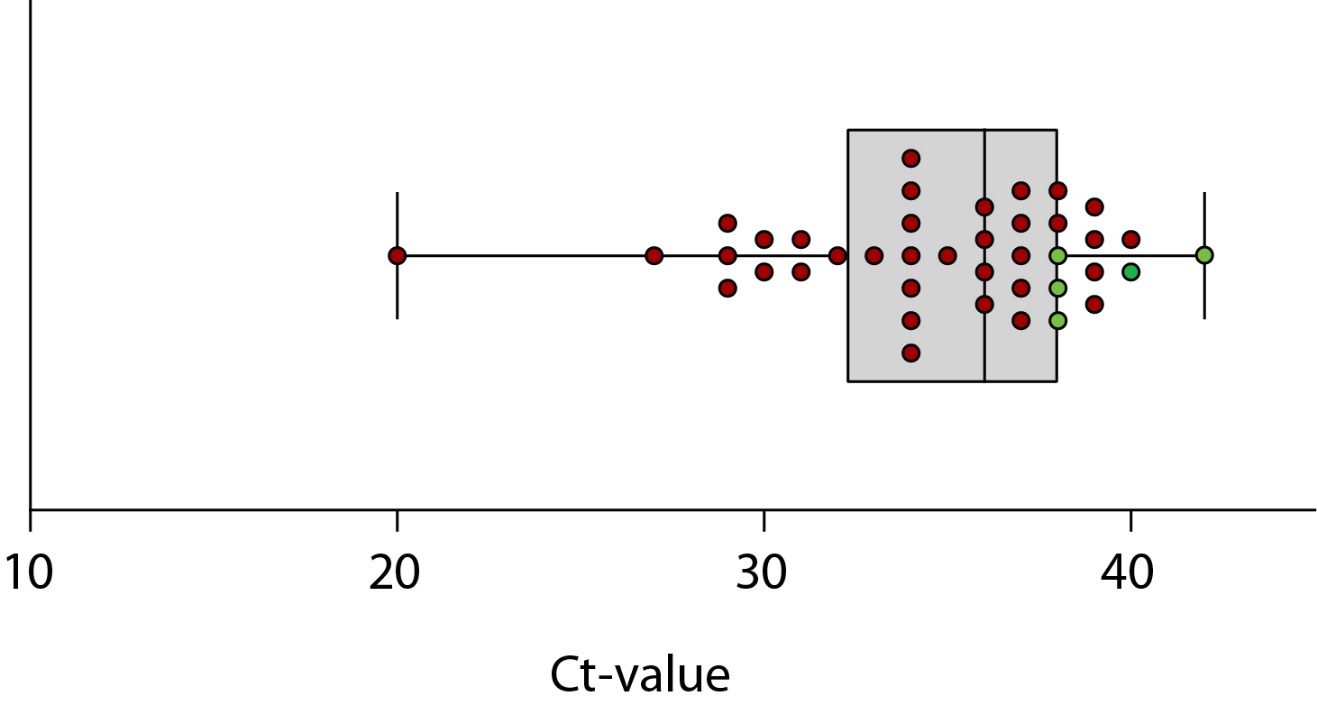
**

**Fig. S2 Phylogenetic analysis of 12 SARS-CoV-2 strains isolated in the B-FAST study**

Genome sequences of 12 SARS-CoV-2 strains isolated in the B-FAST study were included in the analysis. Seven strains derived from isolates detected in school 5 (high burden school Cologne) (sequence IDs: K-5750, K5744, K-5748, K-5753, K-5739, K-5740, K-5747, K-5747 – **green label**). Four strains were isolated in positive individuals detected in primary school 2 in Duesseldorf (D-996, D-997, D-998, D-1000 – **green label**). For comparison analyses 40 additional SARS-CoV-2 genome sequences were included. 32 sequences were obtained from randomly selected patients tested positive for SARS-CoV-2 at the University Hospital Cologne during November and December 2020 (**blue label**). Eight additional sequences derived from strain pairs identified in four cluster outbreaks in the Cologne region (**Orange, pink, purple and red labels; pairs 1-4**).

Pairwise genetic distances

- Median pairwise distance within the whole dataset was 0.0009 (max=0.0014; min=0.0000).
- Median pairwise distance within the school study dataset was 0.0008 and max=0.0011. The distance between the sequences K-5744 and K-5748 was 0.0000 (siblings).
- Heterogeneity analysis within the eight control isolates showed higher variability, with a maximal pair distance of 0.0014 and an overall median distance of 0.0011.
- Heterogeneity analysis within the paired cluster outbreak isolates showed minimal variability, with pairwise distances of 0.0000 for all 4 pairs of sequences.

Based on this analysis, we detected infections with identical isolates in B-FAST study participants K-5744 and K-5748. These two children are siblings and transmission most likely occurred in the respective household. All other strains isolated from B-FAST participants were genetically distant. This is also the case for strain pairs detected in the same class (Class 10E, class 4B and class 3A) (see **Figure S2** below).

**Fig. S2: phylogenetic analysis of 12 SARS-CoV-2 strains isolated in the B-FAST study**


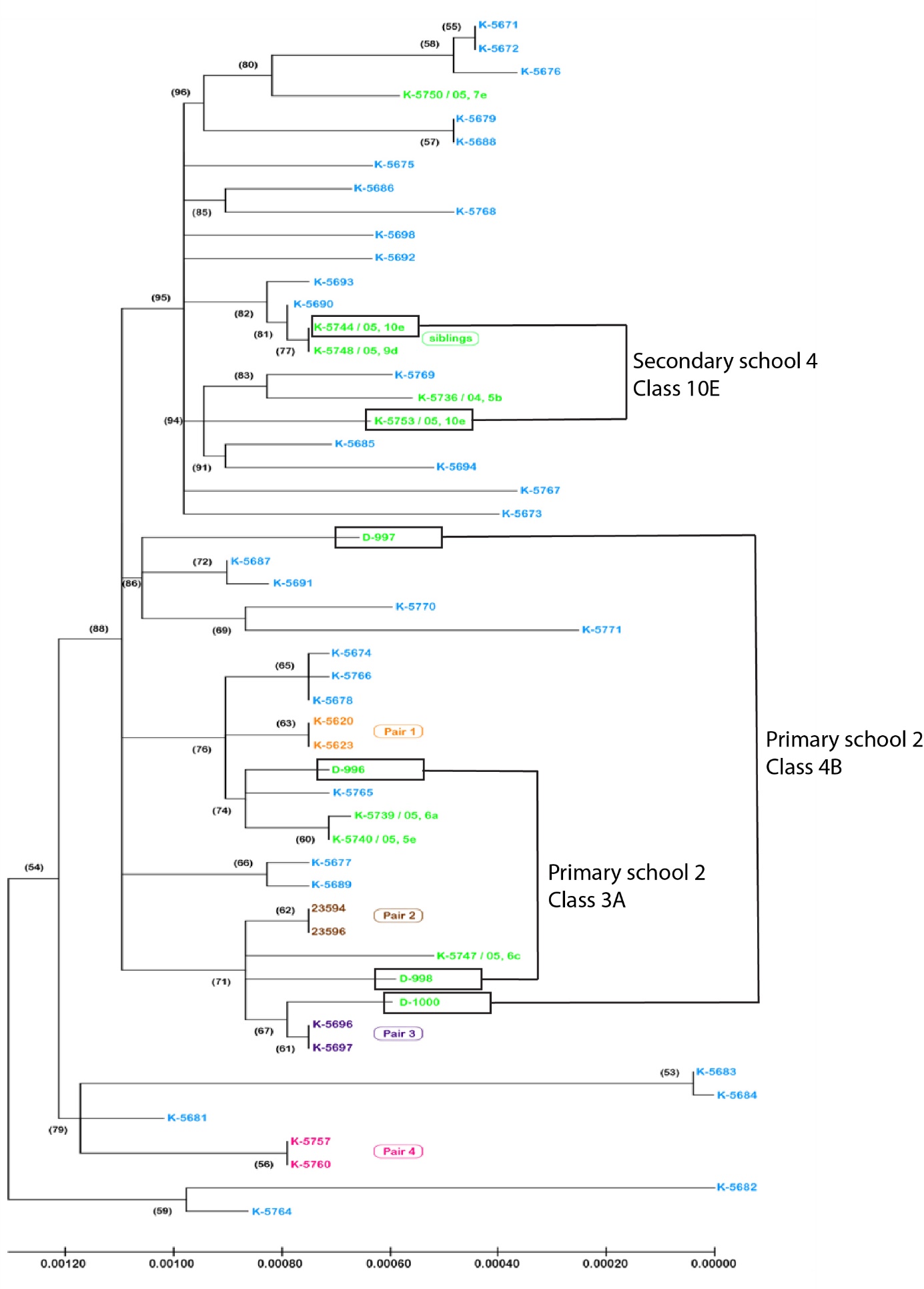


**Table S1: Student participation by school**

| **School** | **Location** | **Student participation**  **n/n eligible (%)** | **Proportion of participants** |
| --- | --- | --- | --- |
| **Primary schools (n=8)** |  | **1,064/1,408 (75·6%)** | **31·3%** |
| Primary school 1 | Duesseldorf | 172/228 (75·4%) | 5·1% |
| Primary school 2 | Duesseldorf | 166/207 (80·2%) | 4·9% |
| Primary school 3 | Heidelberg* | 33/48 (68·8%) | 1·0% |
| Primary school 4 | Heidelberg* | 53/79 (67·1%) | 1·6% |
| Primary school 5 | Homburg Saar | 207/262 (79·0%) | 6·1% |
| Primary school 6 | Cologne | 148/186 (79·6%) | 4·4% |
| Primary school 7 | Cologne | 151/174 (86·8%) | 4·5% |
| Primary school 8 | Munich | 134/224 (59·8%) | 4·0% |
| **Secondary schools (n=6)** |  | **2,322/3,458 (67·1%)** | **68·6%** |
| Secondary school 1 | Heidelberg* | 432/651 (66·4%) | 12·8% |
| Secondary school 2 | Homburg Saar | 221/330 (67·0%) | 6·5% |
| Secondary school 3 | Homburg Saar | 535/659 (81·2%) | 15·8% |
| Secondary school 4 | Cologne  (*District A – Fig. 5c; Table S7*) | 346/758 (45·6%) | 10·2% |
| Secondary school 5 | Cologne  (*District B – Fig. 5c; Table S7*) | 586/698 (84·0%) | 17·3% |
| Secondary school 6 | Munich | 202/362 (55·8%) | 6·0% |
| **Total schools (n=14)** |  | **3,386/4,866 (69·3%)** | **100%** |

*And surrounding Rhein-Neckar county

**Table S2: Baseline characteristics by school type**

|  | **Primary school** | **Secondary school** | **Total** |
| --- | --- | --- | --- |
| **School level** |  |  |  |
| Number of schools | 8 schools | 6 schools | 14 schools |
| **Class level** |  |  |  |
| Total classes in study | 67 classes | 158 classes | 225 classes |
| Classes per school;  median (range) | 8 classes  (3–16) | 28.5 classes  (15–31) | 13.5 classes  (3–31) |
| Class size;  median (range) | 22 students  (8–29) | 23 students  (9–31) | 22 students  (8–31) |
| **Individual level** |  |  |  |
| **Students (participants)** | 1,064 | 2,322 | 3,467 |
| Age [years] |  |  |  |
| Median (Range) | 8 (5–12) | 13 (9–21) | 12 (5–21) |
| Not provided | 0 | 3 | 3 |
| Sex; n (%) |  |  |  |
| Male | 514 (49·8%) | 1072 (47·4%) | 1586 (48·1%) |
| Female | 519 (50·2%) | 1189 (52·6%) | 1708 (51·9%) |
| Not provided | 31 | 61 | 92 |
| **Staff members (participants)** | 222 | 362 | 584 |
| Age [years] |  |  |  |
| Median (Range) | 43 (18–82) | 44 (16–69) | 44 (16–82) |
| Not provided | 5 | 1 | 6 |
| Sex; n (%) |  |  |  |
| Male | 44 (19·8%) | 121 (33·8%) | 165 (28·4%) |
| Female | 178 (80·2%) | 237 (66·2%) | 415 (71·6%) |
| Not provided | 0 | 4 | 4 |

**Table S3: Participation at class level* for students and school level for staff**

|  | **Primary school** | **Secondary school** | **Total** |
| --- | --- | --- | --- |
| **Student participation per class** |  |  |  |
| Number of participants per class; median (range) | 17 students (4–26) | 14 students (1–28) | 15 students (1–28) |
| Participation rate per class;  median (range) | 75·9%  (30·8%–100%) | 68·4%  (3·3%–100%) | 72·0% (3·3–100%) |
| **Participation of staff** |  |  |  |
| Eligible members | 237 | 417 | 685 |
| Members participating; n (%) | 222 members (93·7%) | 359 members (86·1%) | 584 members (85·3%) |
| Number of participants per school; median (range) | 28·5 members  (11–50) | 70·5 members  (55–87) | 45 members  (11–87) |
| Participation rate per school;  median (range) | 95·7%  (63·6% –100%) | 89·2%  (70·0% –100%) | 94·1%  (63·6% –100%) |
| * Student participation at school level is provided separately for each school in table S1 | | | |

**Table S4: Study conduct at class level**

|  | **Primary school**  **(67 classes)** | **Secondary school**  **(158 classes)** | **Total**  **(225 classes)** |
| --- | --- | --- | --- |
| **Duration of testing period** |  |  |  |
| As planned (6 or 9 pooled tests in 3 weeks) | 59 classes (88·1%) | 123 classes (77·8%) | 182 classes (80·9%) |
| Planned follow-up into week 4 or 5 because of positive tests | 4 classes (6·0%) | 7 classes (4·4%) | 11 classes (4·9%) |
| Premature termination in 3^rd^ week due to nationwide school closures* | 4 classes (6·0%) | 28 classes (17·7%) | 32 classes (14·2%) |
| **Number of pooled tests conducted;  median (range)** |  |  |  |
| 2×/week strategy | 6 tests (4–8) | 6 tests (2–8) | 6 tests (2–8) |
| 3×/week strategy | 9 tests (6–14) | 8 tests (5–12) | 9 tests (5–14) |
| **Students per pooled test;  median (range)** |  |  |  |
| one pooled test strategy | 17 students (6–26) | 14 students (2–26) | 15 students (2–26) |
| two pooled tests strategy | 9 students (2–15) | 8 students (1–16) | 8 students (1–16) |
| Total | 10 students (2–26) | 10 students (1–26) | 10 students (1–26) |
| * One primary and one secondary school affected | | | |

**Table S5: Results of individual RT-qPCR testing for students**

|  | **Primary schools** | | **Secondary schools** | |
| --- | --- | --- | --- | --- |
| **Cases with positive samples; N/Total (%)** | | | | |
| Students affected | 7/1,064 students | | 29/2,322 students | |
| Of those, detected on the first day of testing | 0/7 students (0%) | | 11/29 students (37·9%) | |
| Classes affected | 6/67 classes (9%) | | 22/158 classes (13·9%) | |
| Schools affected | 4/8 schools (50%) | | 5/6 schools (83·3%) | |
| **Comparison of sampling technique** | | | | |
| Sampling technique | Saliva swab (lolli) | Buccal swab | Saliva swab (lolli) | Oropharyngeal swab |
| Positive individual samples | 5/512 (1·0%) | 2/552 (0·4%) | 15/1,177 (1·3%) | 14/1,112 (1·3%) |
| Model based odds ratio with 95% CI | 2·7 (0·5–15·5) ^†^ | | 1·1 (0·5–2·5)* | |
| * estimate from mixed logistic model with random intercept for school and class (ICC 0·35), and sampling technique as fixed effect;  ^†^ similar model as for secondary schools omitting the school intercept (ICC for classes only 0·78) because the small number of events did not allow to include it.  CI, confidence interval; ICC, intra-class correlation | | | | |

**Table S6: Results staff members**

| **Facility** | **Occupation** | **Age** | **Sex** | **Day of detection** | **Ct value** |
| --- | --- | --- | --- | --- | --- |
| Secondary school 6 | teacher | 40 | male | Testing day 10 | 32 |
| Primary school 8 | teacher | 29 | female | Testing day 15 | 19 |
| Secondary school 4 | teacher | 46 | female | Testing day 18 | 34 |

**Table S7: Exemplary socio-economic factors of schools in different districts of the city of Cologne** (2019 – districts statistics, city of Cologne, Department of urban development and statistics)

| **Factor** | **District A (high burden school)** | **District B (low burden school)** | **Entire city** |
| --- | --- | --- | --- |
| Population with migrant background (%) | 72·4 | 37·4 | 40·0 |
| Persons living in one household (n) | 2·34 | 1·42 | 1·88 |
| Living space per inhabitant (m²) | 31·6 | 43·7 | 39·3 |
| Government subsidised housing (%) | 17·7 | 1·7 | 6·8 |
| Social benefit claimants (%) | 27·3 | 5·9 | 12·4 |
| Unemployment rate (%) | 12·8 | 5·1 | 7·6 |
| Students attending higher secondary schools (%) | 23·7 | 48·2 | 40·5 |

**Table S8: Classes with possible onward transmissions** (results of the phylogenetic analysis can be found in Figure S1)

| **Event** | **Facility** | **Class** | **Day of detection,**  **first case** | **Day of detection, second case** | **Phylogenetic analysis performed** |
| --- | --- | --- | --- | --- | --- |
| 1 | Primary school 8 | 3 | Testing day 15 | Testing day 22 | no |
| 2 | Primary school 2 | 4 | Testing day 5 | Testing day 12 | yes |
| 3 | Primary school 2 | 3 | Testing day 8 | Testing day 15 | yes |
| 4 | Secondary school 3 | 6 | Testing day 1 | Testing day 9 | no |
| 5 | Secondary school 4 | 6 | Testing day 4 | Testing day 10 | no |
| 6 | Secondary school 4 | 10 | Testing day 3 | Testing day 15 | yes |

**Table S9: Information about 16 out of 17 positive cases at a high burden school in Cologne/Germany, generated via telephone interview with caregivers**

| **Case** | **Symptoms** | **Symptoms onset** | **Chain of infection** |
| --- | --- | --- | --- |
| 1 | Coughing | +1d | Unknown |
| 2 | - | - | Unknown |
| 3 | Fatigue | +1d | Unknown |
| 4 | Headache | -9d | Family |
| 5 | - | - | Unknown |
| 6 | - | - | Family |
| 7 | Headache | +1d | Family |
| 8 | - | - | Family |
| 9 | Coughing, common cold, sore throat | +-0 | Unknown |
| 10 | Coughing, sore throat, fatigue, melalgia | +1d | Unknown |
| 11 | - | - | Family |
| 12 | - | - | Unknown |
| 13 | Coughing | +1d | Family |
| 14 | Headache, melalgia | +1d | Family |
| 15 | Loss of gustatory and olfactory sense | -5d | Family |
| 16 | Headache, sore throat, melalgia, coughing, common cold, fever, loss of gustatory and olfactory sense | +2d | Unknown |

“-“: No symptoms occurred two weeks before and two weeks after positive individual test

“+-Xd”: days after/before positive individual test result.

Family: at least one family member with onset of symptoms and consecutive positive test before positive individual test of study participant

**References Supplementary Appendix**

1. R&D DP, Farr B, Rajan D, et al. COVID-19 ARTIC v3 Illumina library construction and sequencing protocol V.5. 2020.

2. Kumar S, Stecher G, Li M, Knyaz C, Tamura K. MEGA X: Molecular Evolutionary Genetics Analysis across Computing Platforms. *Mol Biol Evol* 2018; **35**(6): 1547-9.

3. Edgar RC. MUSCLE: multiple sequence alignment with high accuracy and high throughput. *Nucleic Acids Res* 2004; **32**(5): 1792-7.
